# Stabbed by motorcycle? Reconstruction of an unusual traffic accident

**DOI:** 10.1007/s00414-022-02941-8

**Published:** 2022-12-22

**Authors:** Holger Muggenthaler, Daniel Bismann, Angelina Autsch, Michael Hubig, Jayant Shanmugam Subramaniam, Gita Mall, Daniel Wittschieber

**Affiliations:** 1 Jena University Hospital – Institute of Legal Medicine, Am Klinikum 1, 07747 Jena, Germany; 2 DEKRA Automobil GmbH, Ziegelhüttenweg 2, 98693 Ilmenau, Germany

**Keywords:** Motorcycle accident, Accident reconstruction, Trauma biomechanics

## Abstract

The reconstruction of traffic accidents involving powered two-wheelers (PTWs) frequently proves to be a challenging task. A case in which a fatal head-on crash of a PTW with a small truck where only minor vehicles damage was observed but resulted in isolated fatal chest trauma is discussed here. External examination of the corpse revealed two lacerations on the back, at the first glance implying sharp trauma. Based on the accident traces, the technical expert assumed an emergency break of the PTW rider resulting in a rotation of the PTW in terms of a wheelie on the front wheel. The first contact between the PTW rider and the tail end of the small truck probably occurred with the upper side of the helmet, and then, the back handle of the PTW caused the stab-like injuries followed by compression of the rider between the small truck or asphalt and the PTW. Based on the few accident traces available, neither a reconstruction of the pre-impact velocity nor a detailed reconstruction of the PTW rider kinematics was possible. However, using an interdisciplinary approach, the principal collision position as well as the injury mechanisms could be reconstructed.

## Introduction

The reconstruction of traffic accidents involving powered two-wheelers (PTWs) frequently proves to be a challenging task [1–3]. Not only diverse injury patterns but also complex pre- and post-collision kinematics may complicate accident analysis. Unlike in car accidents, PTWs are prone to becoming unstable prior to the collision due to steering or breaking maneuvers [4] which in turn can cause diverse injury patterns [5]. Moreover, during the collision and post-collision phases, a separation of the motorcyclist from the motorcycle takes place frequently, resulting in different trajectories and end positions. Further uncertainties arise from the estimation of the energy equivalent speed (EES) and of the change of velocity, which is difficult especially for PTWs [6, 7]. In practical casework, acceptable reconstruction results can be achieved if all traces, injuries, and connecting facts are considered.

We present a fatal head-on crash of a PTW with a small truck resulting in an uncommon injury pattern and only minor damage to the vehicles involved. External examination of the corpse revealed two conspicuous lacerations on the back implying sharp force trauma. At first glance, these injuries were suspected to be from two knife stabs. Hence, in order to exclude homicide, the department of public prosecution aimed at the full reconstruction of the incident by forensic autopsy as well as by additional technical and biomechanical case assessments.

## Case report

A 57-year-old rider of a PTW (Yamaha Tracer900 GT) collided with the tail end of a small truck (Volkswagen T4), which initiated a left-turning maneuver. The final positions of the PTW and the PTW rider were next to the point of collision, with the PTW rider in a supine position and his legs under the PTW. There were no relevant deformations of the motorcycle and only moderate deformations of the rear door of the truck. The accident happened on an urban road with a speed limit of 30 km/h. According to the technical expert, the gearshift of the PTW was in the fifth position.

**Fig. 1:**
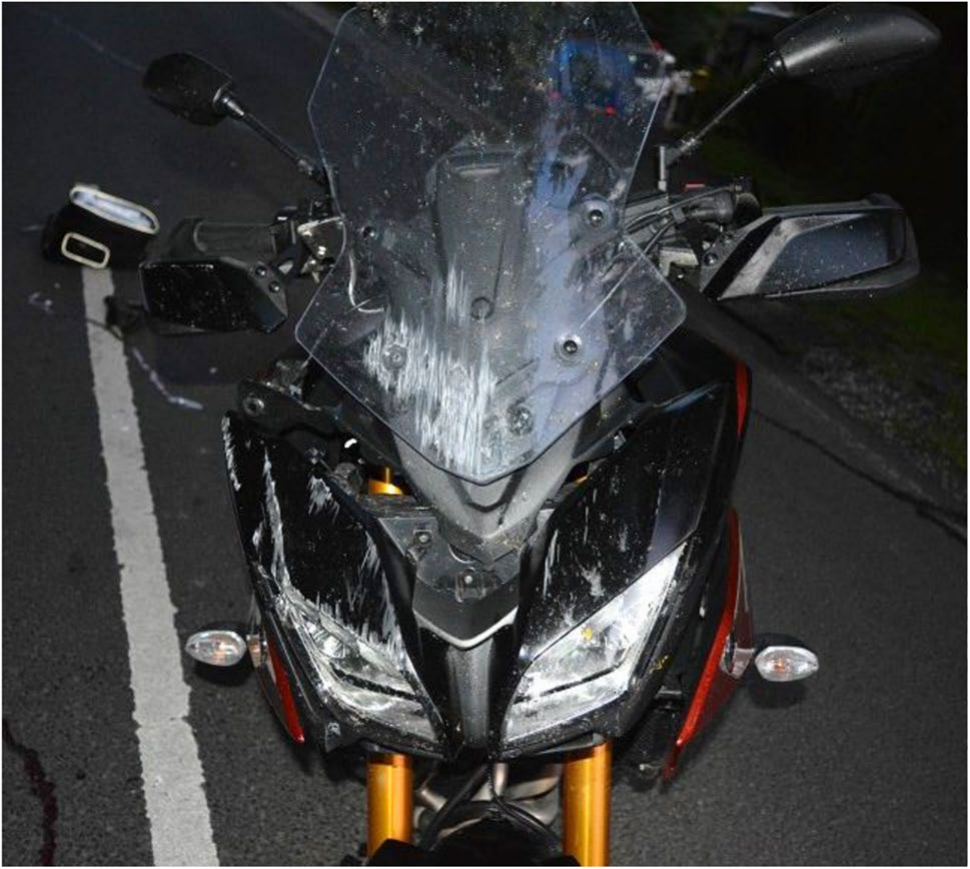
Scratches of the front shield

**Fig. 2:**
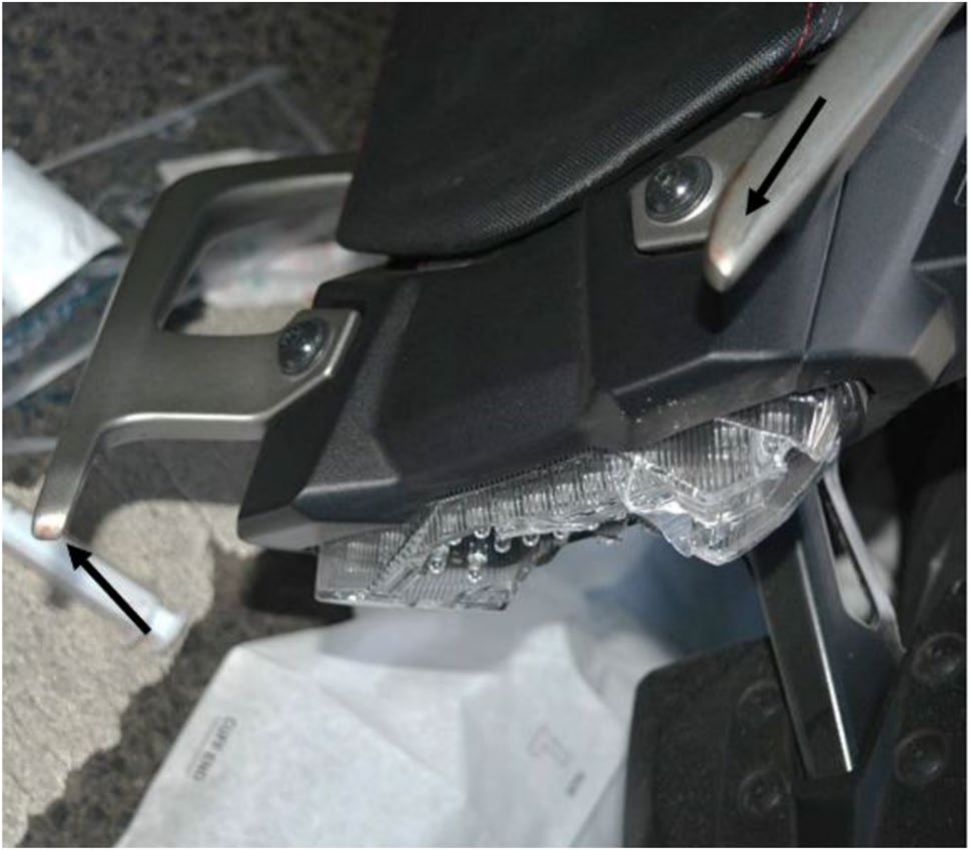
Broken PTW backlight and the two back handles with blood marks (arrows)

**Fig. 3:**
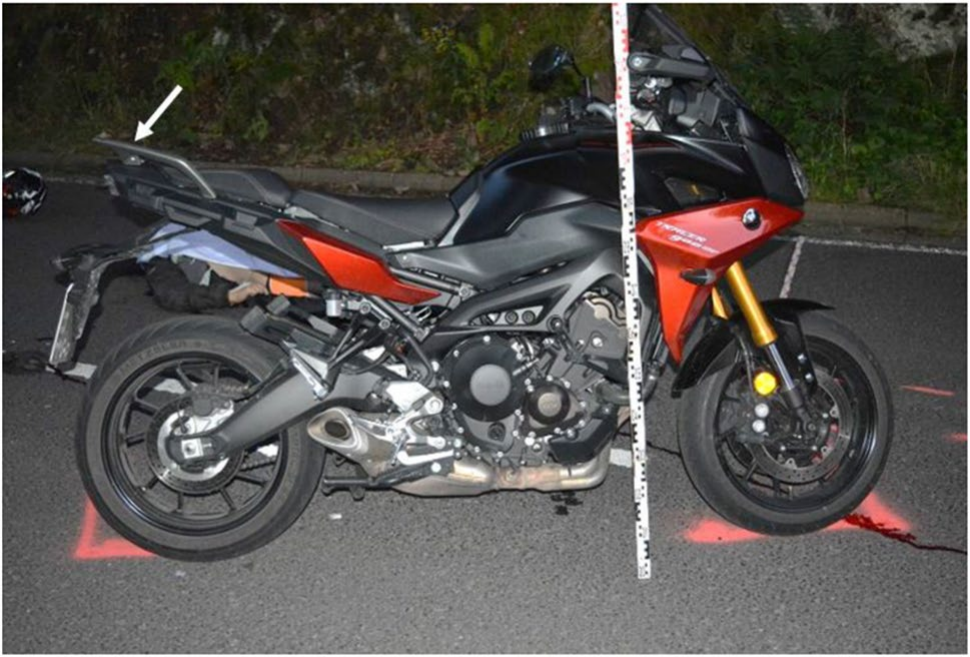
PTW overview, back handles on the tail end of the PTW (arrow)

**Fig. 4:**
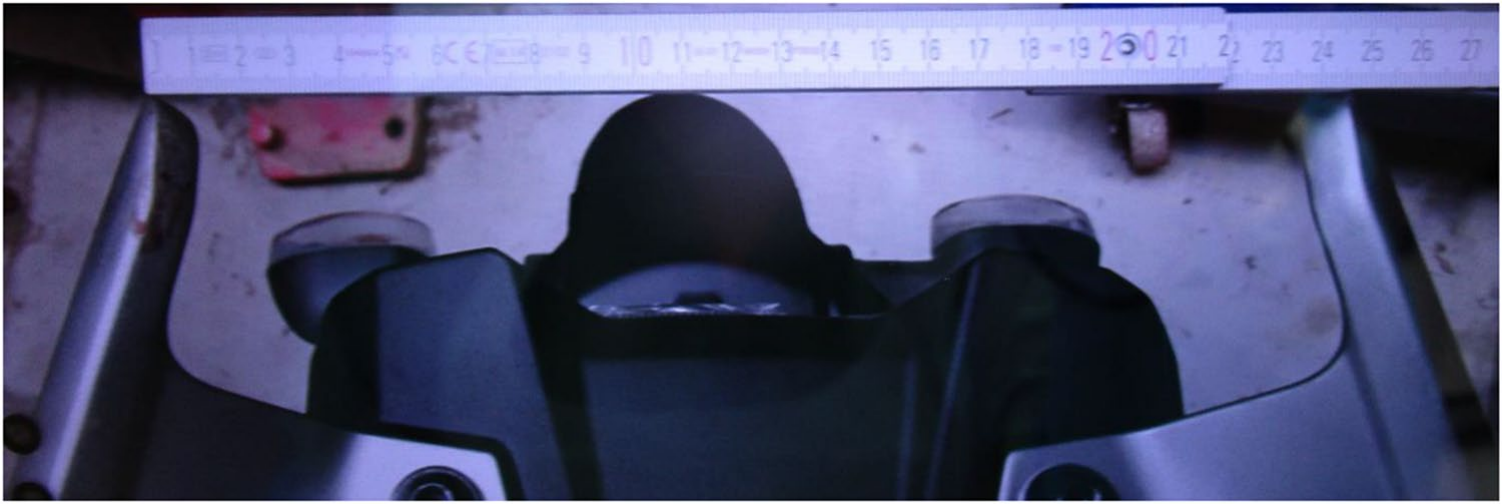
Photograph of the back handles, taken by the police officer using a smart phone

**Fig. 5:**
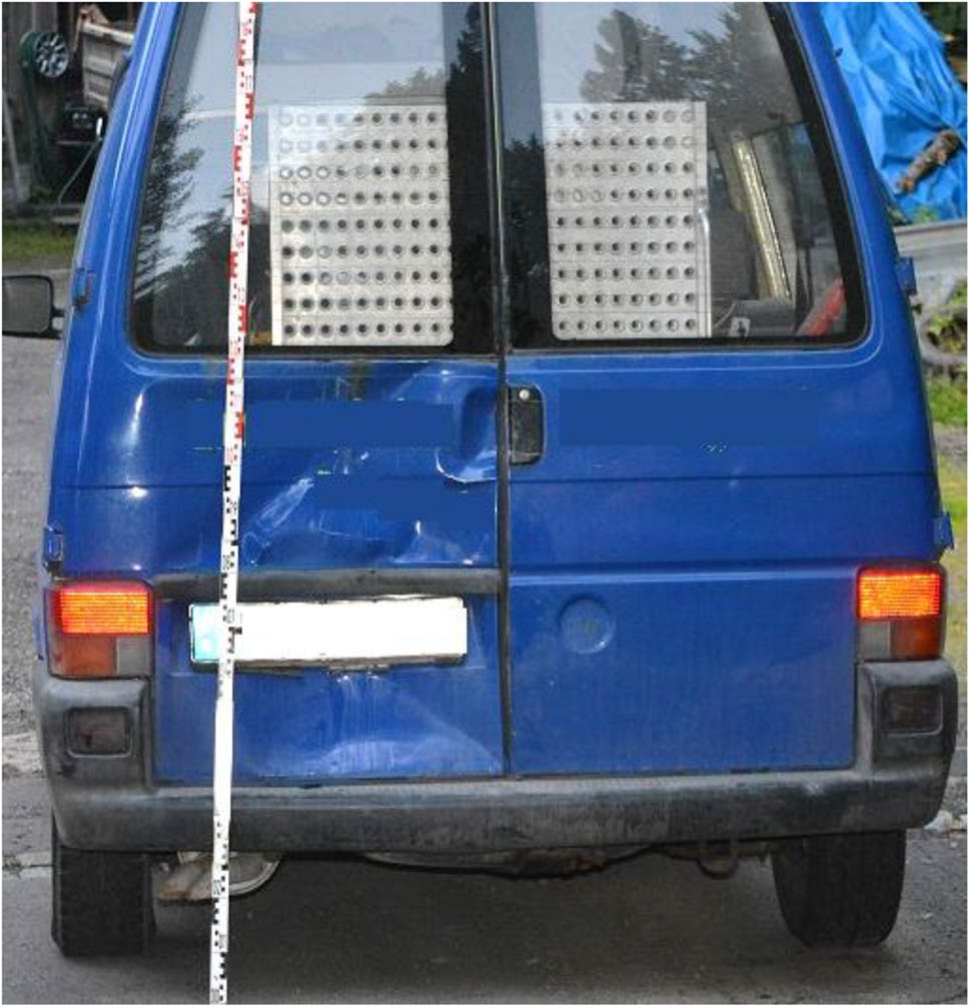
Deformations of the left rear door

**Fig. 6:**
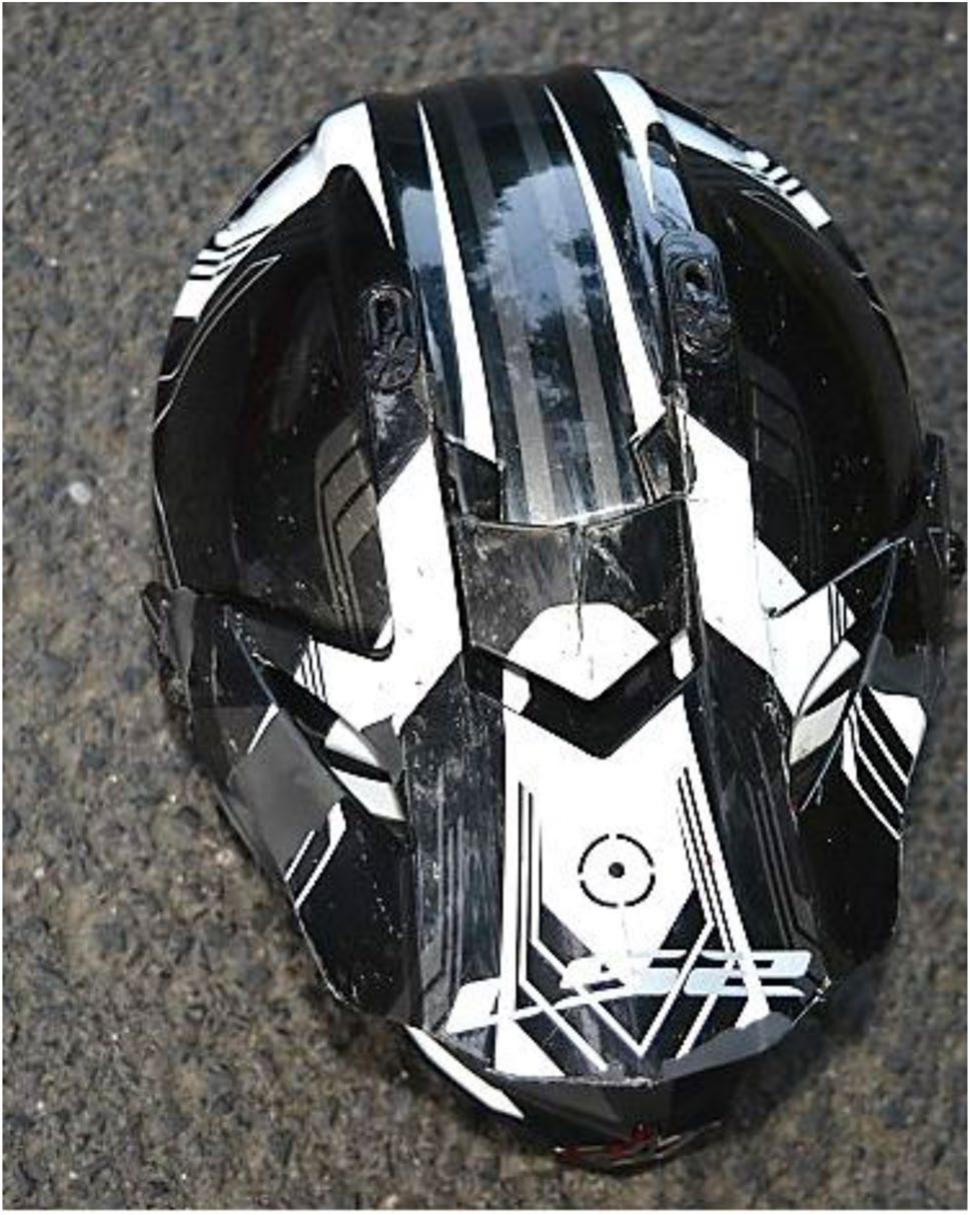
Scratches on the top of the helmet

Figure 1 shows scratch marks of the PTW front shield without deformations of the fork or the chassis. The backlight of the PTW was broken, and the back handles were marked with blood (Figs. 2 and 3). According to a photo taken by a police officer (Fig. 4), the distance between the distal ends of the handle bars was about 24.5 cm. Moderate deformations of the left rear door of the small truck are depicted in Fig. 5. Longitudinally oriented scratch marks on the top of the helmet are illustrated in Fig. 6.

## Autopsy findings

The forensic autopsy of the 57-year-old man (body weight: 110 kg, height: 190 cm) was carried out 4 days after the incident and revealed the following injury pattern:

- Two transversal fractures of the sternum
- Multiple rib fractures on both sides with serial fractures in the anterior (right) and mid-axillary (left) line as well as paravertebrally (bilaterally)
- Bilateral lung lacerations (due to rib fractures)
- Laceration of the pericardium
- Fractures of the 2nd thoracic vertebral body and between the 5th and 6th thoracic vertebral bodies
- Fractures of both scapulae
- Lacerations of the aorta ascendens and within the aortic arc
- Extensive hematoma of the dorsal soft tissues
- Two skin wounds on the upper back with nearly sharp wound edges and a depth of circa 3 cm
- Fracture of the lumbar spine between the 1st and 2nd lumbar vertebra
- Abrasions and avulsions corresponding to both patellae
- Superficial abrasions on the hands and in the face
- Signs of exsanguination

**Fig. 7:**
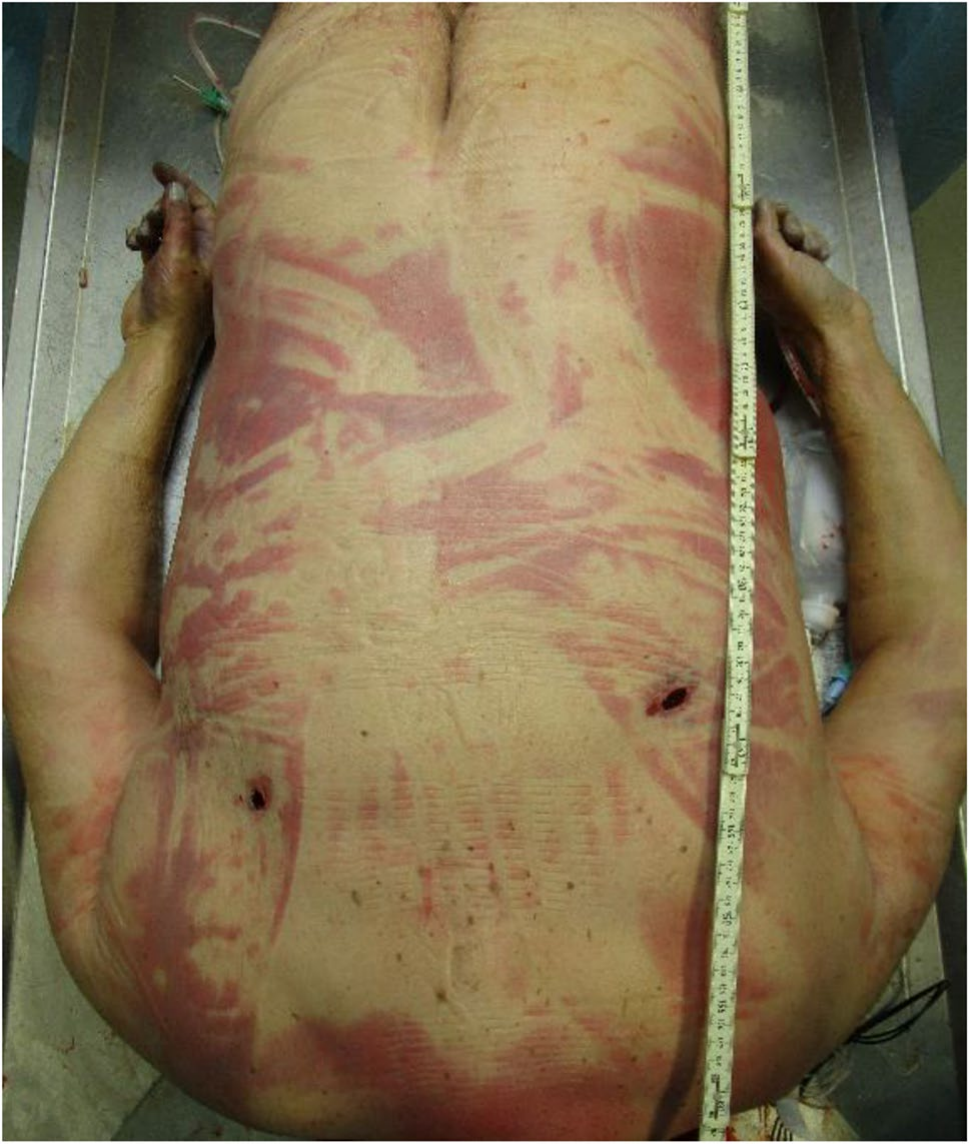
Two stab-like wounds on the upper back

**Fig. 8:**
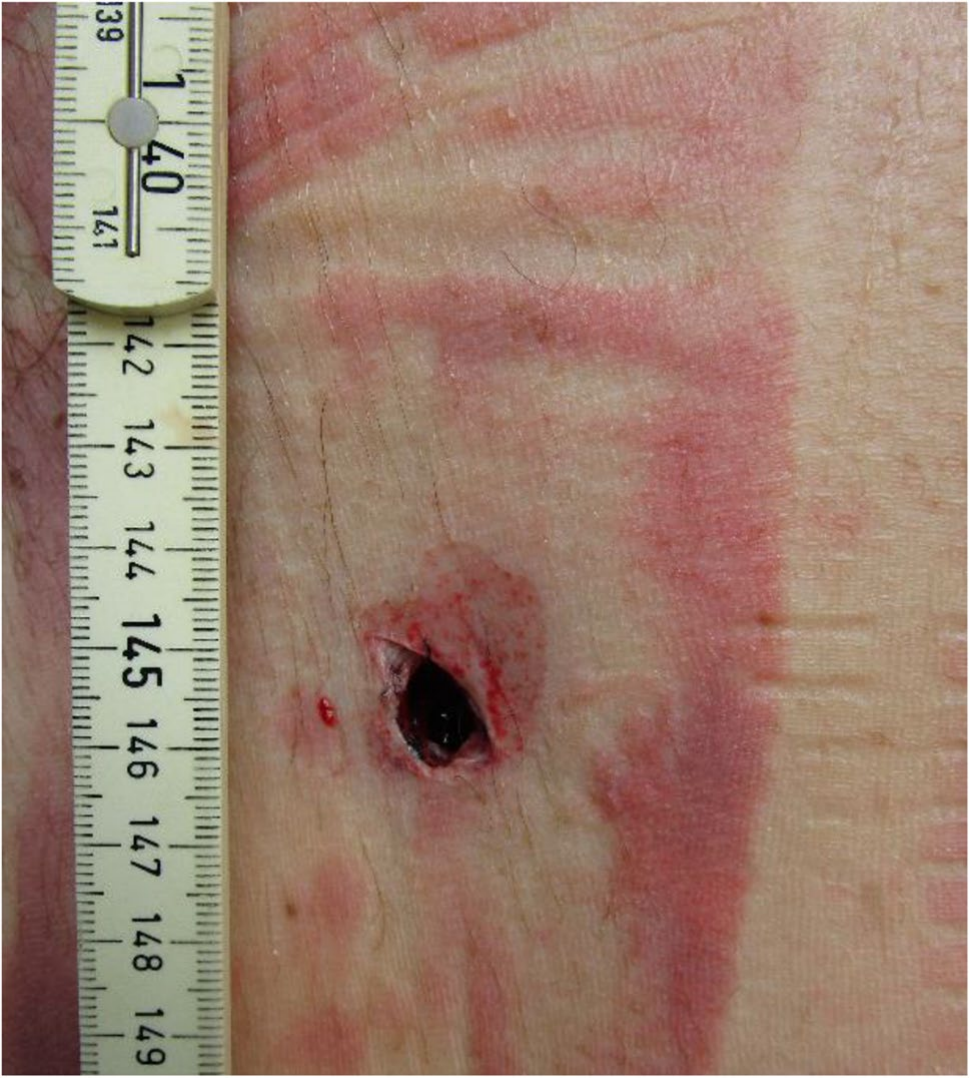
Stab-like wound on the right upper back, in detail

Figures 7 and 8 show the nearly sharp-edged lesions on the upper back of the PTW rider. The wounds were between 2 and 3 cm in length and penetrated the deeper back soft tissues. With respect to the ground level, the wounds were found at a height of 137 cm and 146 cm, respectively. The horizontal distance between both lesions was 27 cm. Corresponding to the skin lesions, the leather biker jacket exhibited two lacerations.

Toxicological analysis did not show any relevant concentrations of alcohol, narcotics, or drugs.

The severe chest trauma was determined as cause of death. Significant organ pathologies that could have affected or caused the incident were not found.

## Material and methods

The iOS based LiDAR scanner of the Apple© iPad Pro 2020, 2nd generation was used for 3D model generation. In [8], the LiDAR technique is described. 3D point clouds as well as the texture were exported to an obj-file. The obj-file was then imported into the accident reconstruction software PC-Crash, version 12.0, where triangulation was performed.

## Accident reconstruction

Head-on PTW collisions normally involve deformations of the PTW fork with corresponding marks and deformations of the opponent car. In our case, we found only slight to moderate deformations of both vehicles, which cannot be explained by a head-on collision between the fork of the PTW and the PTW rider with the tail end of the small truck. Furthermore, the injury pattern with the characteristic skin wounds on the back of the PTW rider was neither consistent with a standard head-on collision. Based on the traces of the helmet and the deformation of the rear door of the small truck without marks of the front wheel of the PTW, we assume the following collision scenario:

Since the PTW was in its 5th gear, we assume a pre-collision speed higher than 30 km/h. Most probably, the PTW rider perceived the braking and turning maneuver of the small truck too late. In order to prevent a collision, he probably initiated an emergency brake resulting in a rotation of the PTW and a wheelie on the front wheel. These rotations can be associated with relatively high rotational velocities of the PTW, while the PTW rider initially maintained in an upright-seated position. The first contact between the PTW rider and the truck probably occurred with the upper side of the helmet. Because of a relatively high rotational moment of the PTW, the back handle would have caused the stab-like injuries to the PTW rider’s back. We assume a subsequent thoracic contact interaction between the PTW and the PTW rider, where the upper body of the PTW rider could have been compressed between the back door of the truck and the PTW or between the asphalt and the PTW.

**Fig. 9:**
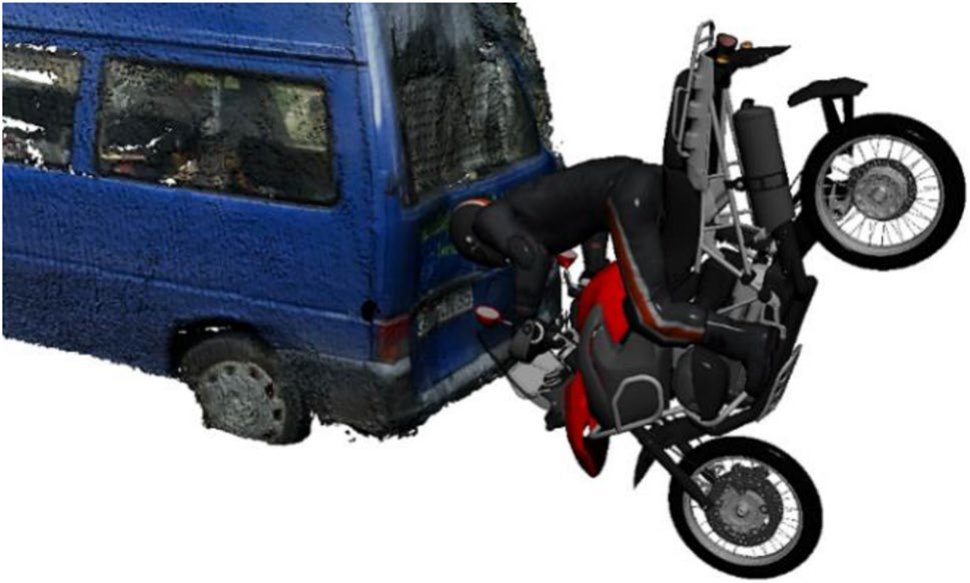
Collision position, which is compatible with the available data. Note that this illustration does not show the type of motorcycle used in the present case

**Fig. 10:**
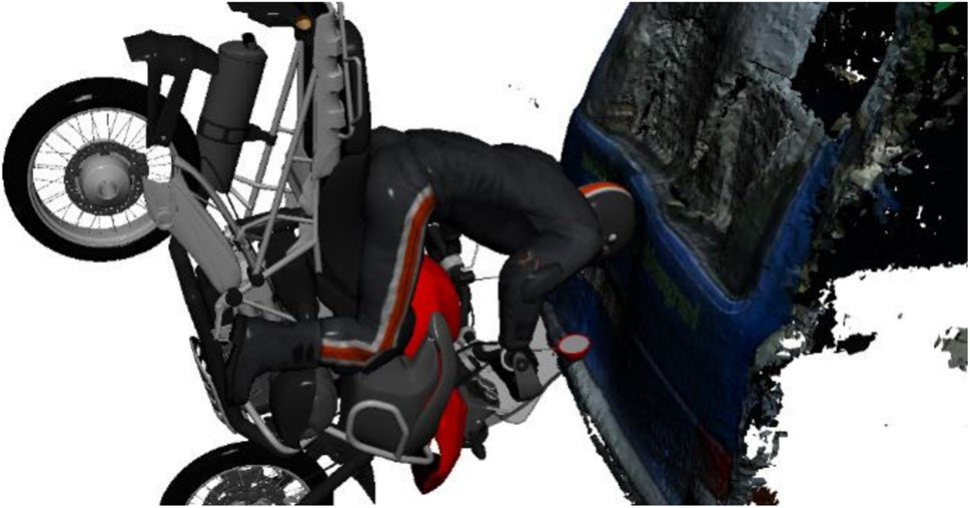
Collision position, head impact

Figures 9 and 10 show a collision position, which is consistent with the damages to the PTW and the small truck. Injuries to the cervical spine did not occur, even though cervical injuries could have been expected in a collision position similar to Fig. 9. Using the data available concerning damage, injury pattern, and final positions, a detailed reconstruction concerning pre-crash velocities, the exact collision position, and the injury pattern was not possible.

## Discussion

In our case, the PTW rider sustained a fatal chest trauma with a bilateral flail chest, several aortic lacerations, lung lacerations, and a pericardial laceration. There were no relevant head injuries and only minor injuries to the extremities. Albeit, PTW accidents often involve a polytrauma with severe thoracic injuries, and an isolated fatal chest trauma is rare. Gidion et al. [9] analyzed motorcyclist injuries in the GIDAS (German In-depth Accident Study) database. Concerning AIS3 + injuries (according to the abbreviated injury scale at least serious injuries), the most frequent ones were femur fractures, rib cage fractures, lung injury, tibia fracture, and brain injury. Considering all PTW accidents including road surface impacts, thoracic injuries were frequent AIS3 + injuries.

Severe chest trauma in PTW accidents is often due to a direct impact with a car or another obstacle, while impacts on the road surface without contacting any other obstacle is not that hazardous. According to Ding et al. [10], the risk of AIS3 + injuries for ground impact cases was less than 25% for a crash speed of 100 km/h, but risk was about 44% for cases with a direct impact on a passenger car. Considering aortic lacerations, Otte et al. [11] found a significant increase in the occurrence of this type of injury in the cumulative distribution of real world accidents for velocity changes higher than 50 km/h. Cavanaugh et al. [12] performed PMHS (post-mortem human subject) sled tests with impact speeds between 24 and 37 km/h. In five out of 17 cases, they found an injury to the aorta.

The two wounds on the back of the PTW rider were caused by an interaction with the back handle bar of the PTW. There was a discrepancy between the distance of the distal ends of the handle bar and the distance of the two wounds, which was approximately 2.5 cm. This discrepancy may be associated to the body posture of the PTW rider during collision. Unlike the situation of the corpse during autopsy, the scapulae could have been closer to each other during the incident due to the muscle tone of the PTW rider sitting on his PTW.

In our case, the data and facts available render speed estimation impossible. However, from the fact that the PTW was in its fifth gear, we assume a pre-crash speed significantly higher than the speed limit of 30 km/h. The considerable injury severity is explained by a collision position similar to that depicted in Fig. 9, where the PTW rider could have been compressed between the small truck and the rotating PTW or between the asphalt and the PTW. The two conspicuous wounds on the back of the PTW rider prove a contact with the PTW handle bar. Extensive blunt injuries of the thorax on the PTW rider can be due to dynamic compression of the PTW rider between the PTW and the small truck or asphalt. The comparably high mass of the PTW exceeding 200 kg causes high contact forces on the PTW rider.

The illustration in Fig. 9 shows a reconstruction, based on the traces found on the helmet as well as on the rear door of the small truck. However, the exact position and posture of the PTW rider on the motorcycle are unknown. From a technical point of view, a certain separation of the PTW rider from the PTW has to be taken into account. On the one hand, such a separation can occur due to a higher rotational velocity of the PTW compared to that of the PTW rider, and on the other hand, actions of the rider like jumping from the vehicle prior to the impact were reported by cyclists [13].

**Fig. 11:**
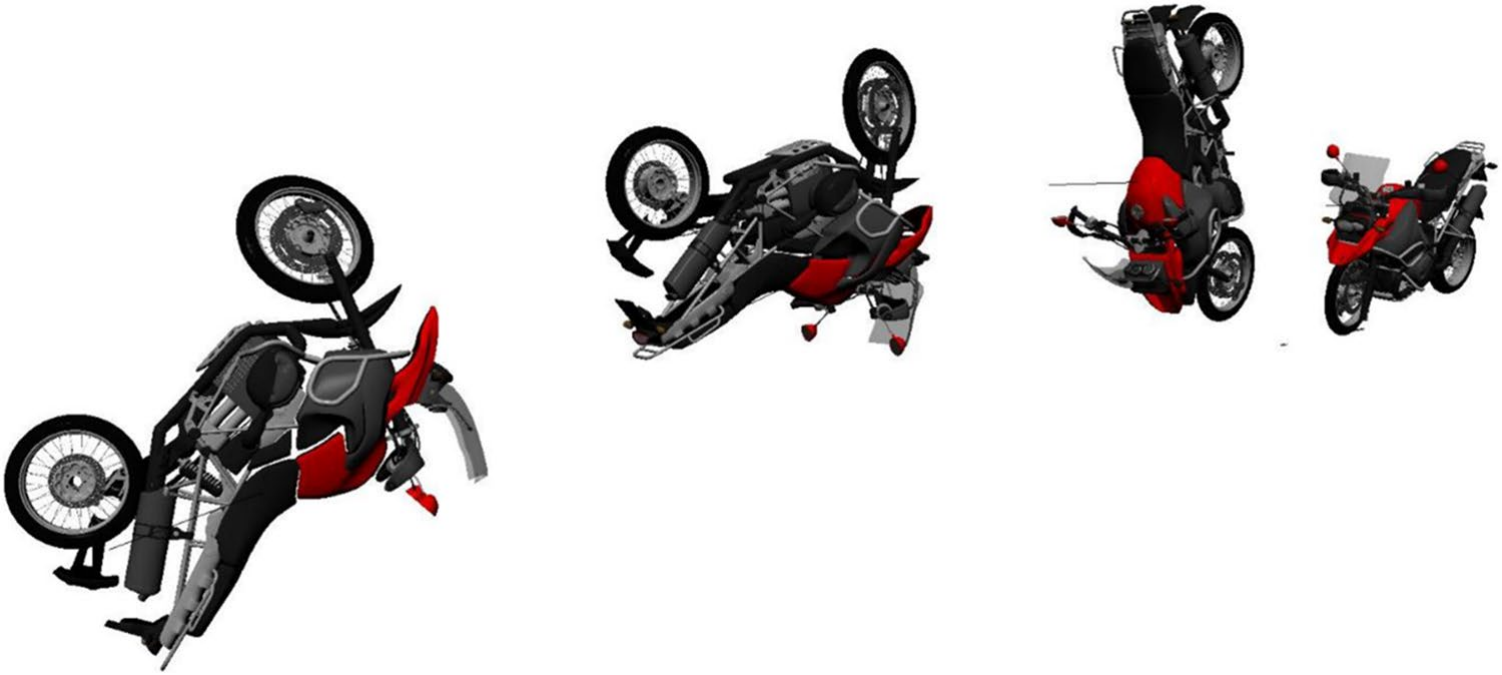
Hypothetical kinematics of the PTW

**Fig. 12:**
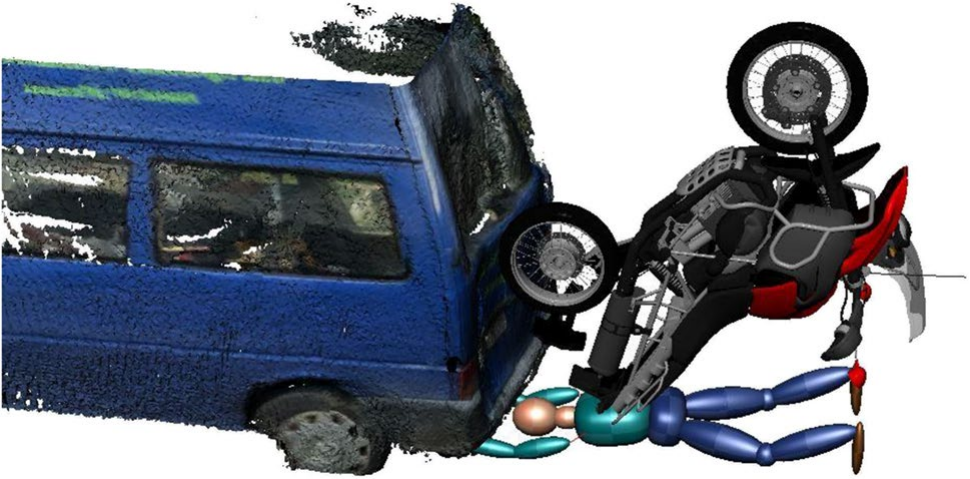
Hypothetical compression of the PTW rider between the PTW and the asphalt

From our point of view, an exact reconstruction of the kinematics is not feasible. Possibly, a separation occurred in a way that the PTW further rotated, while the PTW rider contacted the back door of the van. A possible kinematics of the PTW is shown in Fig. 11. Figure 12 depicts a hypothetical scenario with a compression between of the PTW rider between the asphalt and the PTW. The scenario in Fig. 12 is just one of several possibilities of how the compression could have occurred. We did not perform a multibody simulation, because the case-specific conditions are far too complex to be modeled using multibody technique. Accordingly, the PTW bitmap as well as the rider model in Fig. 12 is positioned without numerical simulation.

The collision position in terms of a front wheelie of the PTW is uncommon. In [14], this scenario is called “End-over, endo, reverse wheelie” and is described as follows: “An extreme forward pitching motion; typically resulting in the rider and the rear frame assembly going over the front wheel in the direction of travel.” In only 6 out of 921 cases with a loss of control of the PTW rider, the authors found such an accident scenario. Brown et al. [15] analyzed causal factors for PTW accidents in which as one of the most common accident causes, they revealed a scenario called “looked but failed to see.” This might also apply to our case. A literature research did not reveal any other reports comparable to our case.

The case presented shows that the reconstruction of PTW accidents is a challenging task. Very different unstable conditions of the PTW and the PTW rider can occur prior to the collision, complicating the technical and biomechanical reconstruction. In cases with only a few accident traces and complex injury patterns, a detailed reconstruction of the pre-crash velocity and the kinematics of the PTW and PTW rider might be impossible. However, the application of an interdisciplinary approach often allows the reconstruction of at least the principal collision position as well as the injury mechanisms.

## Data Availability

Not applicable.
